# “I Have No Capacities That Can Help Me”: Young Asylum Seekers in Norway and Serbia – Flight as Disturbance of Developmental Processes

**DOI:** 10.3389/fpsyg.2021.786210

**Published:** 2022-01-05

**Authors:** Sverre Varvin, Ivana Vladisavljević, Vladimir Jović, Mette Sagbakken

**Affiliations:** ^1^Department of Health Sciences, Oslo Metropolitan University, Oslo, Norway; ^2^Faculty of Philosophy, University of Priština (Kosovska Mitrovica), Pristina, Serbia; ^3^Center for Rehabilitation of Torture Victims, IAN, Belgrade, Serbia

**Keywords:** young asylum seekers, flight, developmental psychology, adolescence, qualitative analysis, traumatization

## Abstract

Most studies on refugee populations are organized around trauma-related issues and focus on explaining pathological factors. Few studies are anchored in general developmental psychology with the aim of exploring normal age-specific developmental tasks and how the special circumstances associated with forced migration can influence how developmental tasks are negotiated. This study is part of a larger mixed method study seeking to identify resilience-promoting and resilience-inhibiting factors, on individual and contextual levels, among asylum seekers and refugees on the move (passing through Serbia) and settled in reception centers in Norway. A strategic sample of 20 adolescent and young adult refugees/asylum seekers during flight in Serbia (10) and after arrival in Norway (10) was chosen from a sample of 178 refugees interviewed in depth in Serbia and at receptions centers in Norway. The sample reflects the focus of this paper, which is to explore adolescent and young adult refugees/asylum seekers’ psychological and social needs and resources during flight to and after arrival in the host country, including how developmental tasks are negotiated. Through qualitative analysis, experiences associated with the developmental changes the participants experienced before, during, and after flight are contextualized. Their sense of self, their relationships with their families and their perceptions of their situation as adolescents or young adults in a highly unpredictable situation are presented in the light of relevant theory and findings from similar refugee studies. All the participants have fled from dangerous and intolerable situations in their home countries. They describe extreme dangers during flight in contexts that are unpredictable and where they feel lonely and unsupported. Most have unmet psychosocial needs and have received little support or help for their mental health issues during flight or after arrival in Norway. Suggestions for interventions and resilience-promoting actions are given based on the findings of the study.

## Introduction

Of the 82.4 million people forced to leave their homes (an increase of 2.9 million people from 2020), around 40 million are underaged (<18) and a substantial number are in transition to adulthood. Available data show that from 1990 to 2020 there has been a radical increase in the number of young migrants (15–24), including refugees, of almost 10 million, and an increasing number arrive as unaccompanied minors (IOM, 2021). A large proportion of refugees is internally displaced (45.7 million), and most of those who have crossed borders reside in neighboring countries. A small proportion, including unaccompanied minors and young adults, try to reach Western and other affluent countries to seek safety and possible asylum.

The present situation is characterized by high-income countries’ attempts to hinder refugees from crossing their borders. This policy of deterrence has gradually become the key element of many countries’ policies toward refugees. Deterrence policy causes prolonged flights, more suffering and mental health problems (Hassan et al., 2016), and a higher risk of death (Borja and Black, 2021).

Many refugees from the Middle East and Central Asia reach northern Europe *via* the Balkan route. The entry point to Europe is often Greece. The geographical location of Serbia makes it a transit hub for refugees taking that route. In March 2016, the EU and Turkey signed an agreement to reduce the number of refugees entering Europe by sending migrants crossing from Turkey into Greece back to Turkey and by increasing border security (Weber, 2016). The endeavor to close the Balkan route has only been partly successful, but it has made the flight more difficult and dangerous (Weber, 2017; Jovic, 2018).

The aim of this article is to explore aspects of development among young refugees during flight and after arrival in reception countries in order to understand their developmental, psychological, and social needs and resources and how they manage developmental challenges. Furthermore, and based on this analysis, we will suggest what conditions and types of intervention may be suitable and possible.

Research among refugees in Europe, North America, and Australia has shown an overrepresentation of mental health suffering among individuals with a refugee background compared to the majority populations (Fazel et al., 2005, 2012; Priebe et al., 2010; Sabes-Figuera et al., 2012; Bogic et al., 2015; Hocking et al., 2015; Hassan et al., 2016), even after many years in the host country (Vaage et al., 2010; Opaas et al., 2020). In addition to war-related trauma preceding flight, many refugees have suffered adverse and potentially traumatic experiences during childhood and adolescence (Opaas and Varvin, 2015). High levels of mental health problems among unaccompanied minors have been documented (Grant-Knight et al., 2015; Ferrara et al., 2016).

Many refugees have been exposed to multiple, cumulative, and prolonged human-caused, potentially traumatic experiences such as childhood abuse, domestic violence, sexual and physical assault, persecution, torture, combat, and other life-threatening experiences. This may lead to a decreased threshold for later development of posttraumatic stress disorder (PTSD) and problems of personality functioning, such as affect dysregulation, relational difficulties, identity disturbance, and problematic behavior (Briere and Scott, 2015). Furthermore, childhood adversities above a certain level predict psychological and physical health problems, reduced prosperity, and premature death (Anda et al., 2006).

Cumulative stress, such as exposure to multiple traumatic events, poses a risk factor for mental health among young refugees, including greater suffering and functional impairment (Stotz et al., 2015). Increased levels of depression among unaccompanied minors have been documented (Keles et al., 2016). Unaccompanied minors have high support needs on arrival in the host country (Vervliet et al., 2014), and their considerable mental and social problems often go unrecognized (Grant-Knight et al., 2015; Ferrara et al., 2016). Furthermore, many show a lack of trust in health services (Majumder et al., 2015).

There is a lack of knowledge about psychosocial resources that may sustain post-resettlement psychological adaptation among unaccompanied minor asylum seekers. Oppedal and Idsoe (2015) showed in a study of unaccompanied minors resettled in Norway that they suffered from high levels of ongoing war-related intrusive symptoms and depression. They were, however, still engaged in adaptation processes normative to youth with immigrant backgrounds in terms of constructing supportive networks and developing culture competence (Oppedal and Idsoe, 2015).

Psychological development is a lifelong process, and being forced to flee, often accompanied by severe traumatization, implies ruptures in developmental processes at all ages, inflicting wounds that can be difficult to heal. There are certain critical developmental phases, where ruptures and upheavals may have more serious consequences, especially when support and help are insufficient or lacking, i.e., early childhood, adolescence, parenthood, and transition to old age. There are also indications that mental health problems increase in these phases for refugees (Vervliet et al., 2013, 2014; Betancourt et al., 2015; Ashwini et al., 2016). Moreover, difficulties in exile may influence developmental paths and especially identity development (Johansen and Varvin, 2019, 2020).

We focus the double perspective on adolescence proposed in Erikson’s psychosocial, epigenic model (Erikson, 1950), where psychobiological development is seen in interaction with the social and cultural frame for this development.

In parallel with the normative developmental tasks of childhood and adolescence, refugees face other specific and unique challenges, especially during flight and in reception centers. These are challenges they usually have not been prepared for and that unfold in situations, where guidance from parents or other elders is usually lacking. In addition to managing new ecological surroundings with often extreme danger during flight, many struggle with the consequences of earlier traumatic experiences, loss, and adversity (Anda et al., 2006; Opaas and Varvin, 2015) which may make it more difficult to cope.

The life of a refugee is characterized by many uncertainties which may render young people especially vulnerable. Horst and Grabska (2015) claim that uncertainty is a basic characteristic of the life of a refugee. They distinguish between radical and protracted uncertainty. Radical uncertainty characterizes more acute situations, for example, during flight, which may be extremely unpredictable due to a lack of information needed to make qualified judgements. Protracted uncertainty characterizes longer phases of the refugee experience, often lasting many years, making it extremely difficult to plan or imagine a future with possibilities (Horst and Grabska, 2015). These uncertainties have obvious negative effects on young people’s ability to endure difficult situations and to activate psychosocial resources (Sagbakken et al., 2020).

Erikson proposed identity formation as the primary developmental task during adolescence and consolidation of identity as important in the young adult phase, where autonomy and the abilities to form intimate relationships and plan education/work are central tasks (Erikson, 1950, 1964; Blos, 1967). He explored nuances and variance in developmental processes and how social, historical, and political contexts interact with personal capacities. Central for identity development is a sense of temporal and contextual integration, often understood as corresponding to what he described as “a subjective sense of invigorating sameness and continuity” (Erikson, 1968, p. 19; Syed and McLean, 2016). Identity, then, can be understood as an integration of the numerous, possibly conflicting, aspects of individual lives.

Adolescence is characterized by profound biopsychosocial restructuring, during which important biological changes produce psychical challenges to be met in a changing sociocultural context (Hauser, 1999). Different contexts offer more or less stable opportunities for the developing identity. Adolescence is the time of the second separation-individuation phase, where many earlier challenges and conflicts in the childhood separation-individuation phase (Mahler et al., 1975) are actualized in a new biopsychosocial context (Blos, 1967). The challenges of immigration, where similar themes need to be reworked, have been characterized as a third separation-individuation phase (Akhtar, 1995).

Adolescence and young adulthood are developmental phases of separation from parents/caregiving persons and the process of individuation as a separate person. The aim is to achieve relative independence, while simultaneously preserving relationships with the family and the community. There are important sociocultural differences in how these developmental tasks are negotiated between community-oriented societies and individualistic societies, where adherence to and identification with the family and the group are stronger in the former (Dajani, 2017). Nevertheless, it is possible to delineate some basic features. It is a time when the individual’s emergent self learns to regulate emotions, establishes more secure inner models for relationships with others, and develops unique, creative ways of meeting challenges and developing capacities that will be useful in their future lives in their cultural setting (Blos, 1967; Akhtar, 1999). Central in this process is a sense of future directedness, a concept of what lays a head, and which profession and role one is to have in the adult sociocultural context. To develop, the young person needs a future perspective on his/her development (Greene, 2021).

Young refugees experience obvious obstacles to normative or culturally adapted adolescent and young adult development. Early trauma directly impacts developmental pathways – psychological and neurodevelopmental – and is a major contributor to ongoing derailment of development and to the establishment of vulnerability to a range of psychological disorders (DeBellis et al., 1999; DeBellis, 2001; DeBellis and Zisk, 2014; Opaas and Varvin, 2015; Opendak et al., 2017; Newman, 2019), including development of PTSD (Allen and Fonagy, 2015). Potentially traumatic experiences during early childhood have also been shown to explain the severity of psychopathology after later traumatization (Opaas and Varvin, 2015).

For the adolescent to develop a stable self with a sufficiently secure identity, certain conditions must be fulfilled. Under normative developmental circumstances, this means a relatively stable ecological environment, including sufficient support, recognition and affirmation from parents and elders and from the community (Bohleber, 1996). For young refugees, there are deficiencies in almost all these dimensions. The sociocultural context is alien and often extremely unreliable and dangerous, they often lack parental support and affirmation, they are exposed to antisocial circumstances that are profoundly dehumanizing, and are often subjected to human rights violations that can be traumatizing (Arsenijevi et al., 2017).

This is a context which often contains both radical and protracted uncertainty (Horst and Grabska, 2015), where normative development is largely impossible, where it is difficult to develop useful strategies for the future, and where resilient strategies are hard to find. Thus, a sense of future possibilities so important for development in these phases is adversely affected (Walg et al., 2020).

Due to the psychological separation from the family, many adolescents and young adults are confronted with feelings of fear, loneliness, abandonment, powerlessness, and inferiority during their refugee experience.

In normative development, the “second individuation phase” (Blos, 1967), starting from the sense of being with the parents, entails a process of separation that progresses from differentiation from the parents to a temporarily narcissistic phase, with a tendency to split relationships into all good or all bad, together with devaluation and idealization of others (Streeck-Fisher, 2019). Eventually, in normative development, this process is overcome by the young person’s reconciliation with themselves and their parents. In parallel, they can integrate sexuality in relationships and rework fantasies of grandeur into career perspectives (Erdheim, 2014).

Cultural propositions provide the individual with dispositions that make it possible to navigate phases of development. These dispositions connect the individual with the wider community and provide ways of understanding, deriving meaning from experiences and finding strategies for action (Dajani, 2017, 2018). The cultural dimension in each group and society builds on tradition and on development of knowledge that makes group life (e.g., family life) and intimate relationships meaningful (Rosenbaum and Varvin, 2007; Varvin and Rosenbaum, 2011). Thus, dislocation represents a double movement whereby the learned capacities may no longer be valid in new situations that may differ radically from wherever the learned capacities are valid. In Bourdieu’s sense, the individual is dislocated from the field in which their habitus (cultural, social, and economic capital) has developed (Bourdieu and Passeron, 1990; Prieur and Sestoft, 2006).

According to Bourdieu, “field” is a multidimensional space, where the agent’s position is determined by their cultural, social, economic, and symbolic capital. Habitus designs the learned capacities of an individual, capacities that make the person feel and act as if at home in a certain field. It is obvious that, from the beginning, the refugee operates in fields, where his/her habitus, the ways to understand and the means to act, are not in tune with the (power) relations in these new fields.

Participants in our study come from different contexts, some of which have been characterized by war and danger for long periods, and they may thus have developed a habitus more suitable for danger during flight than those who come from more peaceful societies, where upheavals occurred more recently. In situations of uncertainty, for example in long, undetermined situations during flight, in refugee centers, in hiding, on arrival or while waiting for asylum application decisions, habitus may radically fail to function as something that can preserve meaning and directedness and can guide actions. Being lowest in the hierarchy dominated by smugglers and police/border guards, refugees’ social capital is too low to make any resistance of importance. Their economic status is constantly challenged because of minimal resources to cover necessities and because thefts and robberies deplete their resources. To the best of our knowledge, few studies exist that examine refugees’ developmental needs and problems during and after flight.

Flight represents a radical break with normative development in peoples’ culture of origin, and the aim of the present study is to explore how refugees manage their developmental, psychological, and social needs and resources during flight and shortly after arrival. In this context, it is important to identify resilience-promoting and resilience-inhibiting factors on individual and contextual levels; that is, to explore which resources and deficiencies influence their lives at different stages in the flight journey.

Our preunderstanding is that their refugee journeys represent radical alterations in expected development, and we expect strong variations in how they cope with ongoing adversities during flight (uncertainty, life-threatening situations, physical abuse, torture, etc.). We expect that their resilience capacities are stretched and that many may develop more resigned ways of life, losing hope, while others may develop resilient functioning. We expect developmental challenges to be extremely difficult to meet in the contexts of flight and exile.

Based on our analysis, we will propose conditions and interventions that may promote resilience and better mental health.

## Methodology

This study presents qualitative data from a mixed method study with participants recruited in Norway and Serbia. Results from this study have been presented in earlier publications (Grøtvedt et al., submitted; Sagbakken et al., 2020). Quantitative data related to traumatic events and indicators of mental health are presented in a separate article (Grøtvedt et al., submitted). Qualitative methodology was used to identify resilience-promoting and resilience-inhibiting factors on both individual and contextual levels for asylum seekers during their flight through Serbia and during their stay at asylum reception centers in Norway.

### Sample

#### Characteristics of the Serbian Sample

The participants in the Serbian part of the overall Serbian study consisted of 100 subjects in total, interviewed in two different locations: the first group was recruited in city parks in Belgrade (*N* = 50), where they made a brief stop before organizing the next stage of their journey. The second group was interviewed in a reception center in Preševo in southern Serbia (*N* = 50), where individuals and families were residing for longer periods of time. Due to differences in the settings, we had only 10 female subjects, all of whom were interviewed in the reception center. In Belgrade, it was not possible to reach any women for reasons that are difficult to evaluate. The Serbian sample was recruited between January and April 2017. Interpreters and researchers approached the participants with oral information about the study, and written consent was obtained. Some participants, after hearing about the study through others, approached us and volunteered to participate.

#### Characteristics of the Norwegian Sample

The participants in the Norwegian part of the overall study consisted of 78 participants recruited between September 2016 and June 2018 at five reception centers for families and single adults in different counties, both urban and rural, the latter located in regions far away from the capital.

To initiate and strengthen the recruitment process, an information meeting about the study was held in all the centers. Participants volunteered in connection with the information meeting or were recruited through center administration and staff.

Participants both in Serbia and in Norway were informed that the research had no link to the asylum application process. Both samples were convenience samples, where participants volunteered for the project after receiving information (Patton, 2014). The majority of the participants in the Norwegian sample came from the Middle East and Central Asia and had fled through Serbia.

The inclusion criteria for the whole group were refugees and asylum seekers during flight who were able (mentally and physically) to conduct an interview and aged above 18 in Norway and above 16 in Serbia. In Serbia, one can give informed consent from the age of 16, in Norway from the age of 18.

#### The Participants in This Part of the Study

We made a purposeful sampling strategy out of the total sample of 178 participants. The participants represented the age groups adolescence and young adults^1^, both of which are important phases in the transition to adulthood (16–22 in the Serbian group, 18–27 in the Norwegian group). Their age was designated based on their declared age, but we indicated cases, where there were discrepancies between declared age and the age assigned to them after age testing was performed on participants in Norway. Twenty subjects – 10 participants from the Serbian context and 10 participants from the Norwegian context – were selected in order to gain the best possible in-depth knowledge of the developmental process in adolescence and in the young adult phase. We thus included one group still on the move (through Serbia) and one group in reception centers after arrival in a host country (Norway). Besides representing adolescents (16–18) and young adults (18–27), they were selected according to the quality of interviews, i.e., good, deep descriptions that concerned the research questions, in order to achieve as high information power as possible (Malterud et al., 2021). Information power depends on the study’s aim (whether it is specific enough to gain information needed in terms of sample size), sample specificity (whether the sample shares enough common characteristics to allow the aim/research question to be studied in depth), whether theory is specified sufficiently so that reflections on theory propositions can be made based on the material, whether the quality of researcher–participant dialog was good enough (the good interviews were selected), and whether the sample is homogenous or diverse. In this case, we ensured variation in both background and actual refugee experience at the same time, given the shared destiny of both groups as refugees/asylum seekers.

We found that having 10 subjects from each context was suitable for an in-depth analysis, enabling us to discover patterns as well as variances in the different stages of people’s flight journeys. This study may lay the groundwork for further research on larger or more specific groups in different contexts both during flight and in host countries.

### Sociodemographic Data

#### Serbian Participants

In this part of the study there were nine males from Afghanistan and one female from Iraq. Ages varied from 16 to 22 years (one was married, and one was engaged in their home country, but traveled alone). The education levels varied from 4 to 13 years.

#### Norwegian Participants

The participants in this part of the Norwegian study were all male. Two were from Afghanistan, four from Syria, two from Iraq, one from Ethiopia, and one from Iran. The ages varied from 17 to 25 years. The four who declared their age as 17 had been determined to be 18 or more after age testing. Their education levels varied from 0 to 12 years.

#### Symptoms

In the quantitative part of the overall study (Grøtvedt et al., submitted), we used the Hopkins Symptoms Checklist (HSCL-25; Mollica et al., 2004), which measures symptoms of anxiety and depression, and the Harvard Trauma Questionnaire (HTQ; Mollica et al., 1992, pp. 111–116), which measures a variety of trauma events as well as emotional symptoms considered to be uniquely associated with trauma. In the overall study, the results indicate high levels of mental distress in both samples of refugees (Serbia: *N* = 100, Norway: *N* = 78). Participants in the Serbian sample reported higher levels of symptoms than the participants in Norway. Moreover, the study found that *female gender*, *low education*, *refused asylum*, *high age*, and *concerns about family* correlated with mental distress among the participants (Grøtvedt et al., submitted).

The samples studied in this article (Serbia, *N* = 10, Norway, *N* = 10) also showed higher levels of symptoms (anxiety and posttraumatic symptoms) in the Serbian group.

### Data Production

A brief, open, semi-structured interview guide was developed. The guide consisted of three main questions created to collect the participants´ narratives describing difficult and relevant pre-flight, flight, and post-flight experiences, including their perceived and experienced quality of life from the time they decided to leave their home until the interviews were conducted. Participants were asked to provide examples of situations, persons, or activities that had an impact on them during their refugee journey. Allowing people to tell their stories may facilitate an understanding of how people construct themselves and their relationships in a time of disruption (Frith et al., 2005). As participants may tend to describe the stressors they experienced in each period, they will be specifically prompted to outline the strengths and resources (resilience factors) they brought to bear on the situation that enhanced their coping, such as “Was there anything (or anyone) that helped you handle the difficulties you are describing?” By posing this type of question, we may identify trajectories of resilience during flight and after arrival in the host country. Similarly, to be able to identify both positive and negative experiences (e.g., the presence or absence of services), participants were asked to provide examples of situations, persons, or activities that influenced well-being, and of perceived access to any services. In this way, the participants’ narratives can provide the basis for a contextualized, contiguous interpretation through their stories of specific situations (Butler-Kisber, 2018). Instruments used for quantitative data analysis have been described elsewhere (Grøtvedt et al., submitted).

#### The Serbian Context

Interviews were conducted with the help of translators. The interviews lasted between 1 and 2 h. The interviews were of varying quality, as participants’ motivation varied. Sometimes cooperation with translators did not work well and the context of conducting interviews in a park during winter made it difficult to conduct long interviews.

#### The Norwegian Context

In the Norwegian context, all the interviews were planned ahead, and lasted between 60 and 150 min. They took place in a separate room/office in the reception centers, in a familiar and undisturbed place, thus ensuring confidentiality and facilitated the quality of the interviews.

#### Both Contexts

Interviews were conducted with the help of authorized interpreters, and the same interpreters were used during the research period to strengthen intersubjectivity between the interpreters and the researchers. If preferred by the participants, interviews were also conducted without interpreters (in Norwegian, if the participants in Norway had learned the language) or in English (both places). All interviews were audio recorded, transcribed, and translated into a language suitable for analysis (Serbian, Norwegian, or English).

Because of the difficult circumstances in the Serbian part of the project, we took care to select interviews that were comprehensive and of better quality for this part of the study.

The research included participant observations and field notes in the selected reception centers. In this way, we could move beyond selective perceptions and discover issues that were overlooked during interviews. This contextual knowledge facilitated a better understanding of what had been expressed during interviews (Fangen, 2010). Spending time with the residents in receptions centers and in city parks contributed to building a rapport and facilitated recruitment as well as informal and formal conversations/interviews.

### The Research Teams

The research team in Serbia consisted of a psychiatrist, a psychologist, and two master students in clinical psychology. The research team in Norway consisted of two researchers with backgrounds from psychiatry and nursing/anthropology, and five master students in the field of nursing.

### Qualitative Analysis

The interview guide was composed of three open questions aimed at allowing the participants themselves to construct their stories. The subsequent data analysis was inspired by the principles of phenomenological analysis of Giorgi (1985) as modified by Malterud (2017), an analytical approach that attempts to understand the meaning of events and interactions within the framework of how individuals make sense of their world.

We developed an analytical approach that relied on five interconnected stages: (a) familiarization, (b) indexing, (c) identification of a thematic framework, in this study, a developmental frame, (d) interpretation and development of preliminary categories, (e) confrontation with existing developmental theory, and (f) reinterpretation of themes, contextualization and development of the final conceptual framework.

The main researchers (in addition to the master students) also conducted interviews. In this way, the familiarization process started during the interview process and was followed by in-depth reading of the interviews. Identification of meaningful units and themes (indexing) was done through reading and re-reading the interviews and was conducted in a collaborative process between the authors. A preliminary network of categories was developed through an interpretive process, clustered around concepts developed based on the research questions for this study. The aims of the research were subsequently confronted with the material and refined. As it was important to see the gathered passages in context, whole interviews and transcripts from participant observations were re-read when considered necessary.

The different backgrounds of the researchers/interviewers allowed for negotiation from different perspectives in the process of interpreting the material, basing our analysis on researcher triangulation.

Identity (how they viewed themselves, how they perceived that others view them), relationships with family members (how they described their relationships with their families, the lack of contact with them, etc.), and conceptions of the future (what plans and hopes they had, how these were represented in their minds) emerged as categories of overriding importance as well as themes related to the contexts in which they were living in at the time of the interview. We arranged the passages relating to these themes in a table to provide us the main basis for the analysis.

### Ethical Issues

The collection and analysis of data from Norway and Serbia were approved by the Norwegian South-East Regional Committee for Medical and Health Research Ethics (2016/651). All refugees in reception centers in Norway have the right to receive help from the mental health services. Refugees at reception centers can be referred to mental healthcare professionals through their general practitioner with assistance from social workers at the centers. All participants with severe mental distress during data collection received immediate help from mental health professionals. In the Norwegian context, all the participants provided written consent.

Interviews in Serbia were conducted with verbal approval by the Commissariat for Refugees and Migration and in collaboration with the Center for Rehabilitation of Torture Victims (CRVT) and the International Aid Network (IAN). CRTV offers mental health and medical services to refugees, and any of them who asked for help could receive it from IAN. All the participants received information about the purpose and aim of the research from the interviewer and gave their written or oral consent to participate. Most of the minors were interviewed in the park. Those who were in the centers had guardians, and both the guardians and the minor refugees were informed about the research and gave their oral consent. None of the participants received any reward for taking part in the study, neither in Serbia nor in Norway. The interviews were anonymous, as participants did not provide names.

The data in this study were stored in anonymized format in a secure database.

## Results

### Danger and Unpredictability: No Possibilities for the Future

All participants described life-threatening conditions as the main reason for their flight. Afghan refugees described family members who were killed (mostly fathers and brothers) and how they themselves experienced lethal danger, especially from the Taliban and Daesh (ISIS). Accounts of life-threatening conditions were also given in the narratives of refugees from other countries, and similar accounts were given by participants interviewed in Serbia and in Norway. However, the participants interviewed in Serbia seemed to have the horrors at home more present in their mind and gave more detailed accounts.

Amir^2^, an 18-year-old boy from Afghanistan, interviewed in the park in Belgrade, told how life in his province in Afghanistan became increasingly difficult. His elder brother, who studied at the university, was abducted by a Taliban commander, and killed. He and his family tried to live their life as normal afterward, but then another family member was killed:

*About 2 years ago, my father was also abducted and killed by the Taliban, and it was the biggest shock for the family. When my father was killed, we no longer had support, neither economically nor spiritually, apart from an uncle, but in a way, we lost hope of continuing to live in Afghanistan, we were in danger* (Interviewed in Serbia).

He was 16 years old at the time, and suddenly lost material support and, most importantly, what he called spiritual support, which can be understood as the necessary guidance for taking his place as a man in his family and in society.

Mohammed, a 16-year-old boy from Afghanistan, gave a detailed description of the situation in Afghanistan and of the experience of unpredictability. People could be killed for no reason. He underlined the importance of feeling safe and of having worth as a human being:

*Many innocent people were killed by the Taliban and the Daesh. That’s how it happens there, you can be innocent and be killed. Because of those problems, we left the country and went somewhere, where we can live peacefully and be respected. An uncle who worked in the military was killed by the Taliban. He was abducted and beaten, the Taliban demanded money for him, we did not have enough to pay, and they killed him. We feel safe here, we feel we are worth something. Nobody touches us. We feel respected. In Afghanistan, we were not allowed to go to school out of fear. Whenever we left the house, we were never sure that we would return safely* (Interviewed in Serbia).

Habdeh, an 18-year-old boy from Syria who fled alone when he was around 14, expressed his reason for fleeing in a few words:

*There was war in Syria. There was killing, beheading, and no school. We are a lost generation* (Interviewed in Norway).

These quotations express a general trend in the material and show that flight represents an escape from a situation of a lack of possibilities for young people. As the last quotation demonstrates, these young people feel that they represent a lost generation, a generation without possibilities for future development.

Rajab (judged by immigration authorities to be 19, but saying he was 18), from a minority persecuted by the Taliban, described both danger and lack of possibilities:

*There was war in my home country, so I had no possibility to go to school. The Taliban are dangerous. It was when they said, yes come with us and you get weapons, and you shall kill people who were innocent, he added. Or if you do not come with us, we will kill you* (Interviewed in Norway).

As indicated in the quote, many of these young refugees decided to leave when they reached a tipping point in the form of a growing sense of being in danger, or a fear that someone in the family might be killed or that the Taliban would physically come to their home to recruit young boys.

Abdullah, 22 years old and from Afghanistan, explained the lethal dilemma he faced in his home country. He had lost his father and mother in the war and was living with a relative who was a member of Taliban. The relative tried to prepare him to be a suicide bomber:

*If we are on the side of the Taliban, we must commit suicide (by fighting) for them. And if we side with the police, the Taliban will also kill us. It was very difficult there; we could not protect ourselves anymore* (Interviewed in Serbia).

For the Afghan refugees, the impossibility of living in their home country was repeatedly underlined. They lived under a terror regime, where “the Taliban” was a gruesome and unpredictable force. The participants described their lives as dangerous, with no possibilities, and where normative development as young people was impossible. Being dangerous even to go outside one’s house or village made it extremely difficult to go to school, to form relationships, develop interests, and find a way to develop one’s identity. Many seemed to have developed an embodied sense of being endangered subjects whose main task was to be on the alert for danger.

The aim of the flight was formulated by several participants as an obligation to help their families. Parents often sold their property and everything they had to invest in their journey. There was thus great pressure from the family at home to succeed; an obligation to help their families but without the necessary support to manage it. Pablo, an 18-year-old boy from Afghanistan, talked about his motives for leaving his home:

*My motive is to go to Europe to study, to start working after graduation, to have a better life, to bring my family and to help my family* (Interviewed in Serbia).

The loss of a significant supporting person was often behind their decision to flee, in a situation, where they had to take responsibility as the eldest son in the family. Sander, a 17-year-old boy from Afghanistan, told how the death of his father made him feel obliged to leave:

*My problem was that I left my country. My father was killed. He was the first in his tribe and was killed by the Taliban. I do not want to tell the long story of how he was killed. Because of my brother and mother, and one sister, I went to Europe to get a job and help my family* (Interviewed in Serbia).

To help the family seemed to be part of their identity. They felt an obligation to take on responsibility as family provider – very much as a future goal. For them, this could justify that they had escaped and left their families in difficult situations.

For all the participants, the flight represented a change in their way of managing life. They had to go through dangerous situations and thus constantly be strong fighters, without the possibility to express their feelings, needs, or sense of helplessness. At the same time, many carried a heavy responsibility for the family they were obliged to help and, hopefully, provide for in the future. The flight thus represented a profound identity-changing experience, an abrupt transition to an expectation of being strong young men with adult responsibilities.

### They Were Not Prepared for What Was to Come

Desperation and hope drove the participants to flee, but few were prepared for what would come during their flight. The descriptions of their flight experiences were filled with danger, terror, deceit, and lost hopes, and resembled situations, where all normative developmental possibilities as young people were put on hold.

Ahmad, 16 years old, from a minority in Afghanistan, stated that he decided to flee because there was no future for him in Afghanistan. However, he was not prepared for all the dangers he would encounter:

*We had a lot of problems along the way, we were scared. We were afraid of the police, of wild animals (in the woods during the escape), that they would lock us in a closed room and that they would ask us for money, as was the case. We had a lot of problems* (Interviewed in Serbia).

Many described being exploited when working during their flight to Europe. Several told of how they had worked hard (in Turkey, for example) but did not get paid or were paid far less than promised, not receiving food and the like. Violence, executed by smugglers but also by police and border guards, was frequent.

Mohammed, a 16-year-old Afghan boy, talked about one of the many arbitrary stays in prison, similar to the experiences of many of the participants:

*There were five or six in each room (in a refugee camp in Bulgaria). The police went in groups, pulled out one and beat him in front of us, as much as they wanted, they beat him* (Interviewed in Serbia).

Another frequently mentioned situation filled with danger was border passing. Abdullah, 22 years from Afghanistan, told how every time he and his friends tried to cross the Bulgarian border the police would steal their few essential valuables and subject them to physical violence and humiliation:

*We tried to cross the border three or four times, and each time we failed we were beaten by the Bulgarian police. Bulgarian border guards took our money and all our valuables. They took sneakers and boots from us; they even took our socks. We were wearing only our undershirts and underwear, and they let us go back. It was very cold, and it was very hard; I just got beaten up and told to go back. On the third or fourth attempt, the police took everything from me, both my boots and my clothes* (Interviewed in Serbia).

Many of the participants had similar experiences every time they crossed a new border; being arbitrarily imprisoned, deprived of all valuables, being threatened, beaten, and left in a powerless position. Pablo, an 18-year-old boy from Afghanistan, described the following experience:

*The Bulgarian police took us to the police station in Sofia, they took our money and phones and beat us more. The Hungarian border guards returned us, but before that they took our money and any valuables that we had* (Interviewed in Serbia).

Several of the participants mentioned how the loss of their mobile phones increased their sense of being powerless and unsafe; not being able to communicate with friends they lost contact with on the way or with parents and relatives in their home country, all of which represented sources of comfort and belonging.

Moreover, the smugglers represented danger in the sense of exposing the refugees to threats, harassment, and violence. Abdullah, a 22-year-old boy from Afghanistan, talked about this additional source of danger:

*They were very dangerous, (the) smugglers. Here and now when I talk about it, I am shaking. Very dangerous people. They physically harassed and beat us, they hurt my head, I even have a scar on my head. I do not know if my arm was broken, but it was red and swollen. I got so beaten on my head that sometimes I feel like I’m not thinking normally. I was beaten so badly in those days that I forgot how much trouble I had in Afghanistan. (…) In one room underground, a very small and dark space, one smuggler beat us every day* (Interviewed in Serbia).

As illustrated above, people with authority and power often use their position to abuse, maltreat, and exploit young refugees, depriving them of everything, from essential means to survive to a sense of human worth.

### Help and Support During Flight

Stories about support, care, and help were sparse, and for many they were totally lacking. Some recounted experiences of getting help during their flight in the Middle East. Arriving in Greece was for some a good experience of being welcomed, helped, and supported. Others told of being helped during their journey through Europe (having train tickets bought for them and being given food). One reported getting help from health care institutions and from family when ill, and some reported how well they were received and helped by the police on arrival in Norway.

Abdullah, a 22-year-old boy from Afghanistan, talked about his experience with Kurds in Iraq, where he not only was helped, but felt supported in a way he felt was empathic:

*Those people, the Kurds, when they saw how poor and miserable we were, they all gave us food, they were very kind. I cannot forget them. The Kurds on the border with Iran asked us where we were from. We said from Afghanistan. They gave us food and I cannot forget that. I also worked in a bakery. They were very kind, they gave us money, they gave us everything. I told my life story to the owner of that bakery. When he heard my story, he paid me more than other Afghans. He told me, “Do not go. Stay here and work for me.” I said I had to go further and get my life in order because my brother and sister-in-law are in a bad shape and I had to help them* (Interviewed in Serbia).

Most of the participants traveled alone or in groups with other refugees of similar age, and they could help and support each other to some degree, as exemplified by Sander, a 17-year-old boy from Afghanistan:

*I have a lot of friends here in the barracks and it is a good experience. I can talk to them. When I am lonely I tell them about my sadness and in some way, I am relieved* (Interviewed in Serbia).

However, when the participants started talking about good experiences, the bad ones often came back to their mind. Mohammed, a 16-year-old boy from Afghanistan, talked about how he felt received in a good way in Serbia before he reverted to a situation filled with violence and danger:

*It is a good experience that we are here, we are satisfied. People from different countries are here, they come from European countries to help us. It is a nice experience. I think that the best people in the world who have seen my eyes are the people of Serbia. (…) The worst experience was when I was in Bulgaria in demonstrations, Camp H. that is the worst. After the demonstration, more than 1,000 policemen came to the camp to beat us* (Interviewed in Serbia).

Most participants said that their flight was characterized by uncertainty in acute situations of danger, but also as unpredictable in the long run as they could not foresee how it would end. Most of the time during their flight through Asian and Middle Eastern countries, they had to manage without any care in the form of emotional support or health services.

The interviews were characterized by a scarcity of descriptions of good experiences which one might expect from young people in this age group, including relationships with the opposite sex. They described scenes that reminded them of war zones, so they had to focus their attention on ways to survive and cope. However, they often expressed what they wanted for the future, such as the possibility to study and be able to help their families.

A cross-cutting theme was concern about and longing for the family. Habdeh, the 18-year-old boy from Syria mentioned earlier, told of how he fled when he was around 14 and spent 2 years alone in Turkey working under bad conditions (long working hours, little payment). He remained in the demanding situation in Turkey, but the situation for his family seemed to worry him the most:

*I am young but I look much older because I have experienced so much (…). You think all the time. I think of my family and how they are. (…) Has something happened to them? How are they? How do they manage? One thinks of them all the time*.

Even if many of these young boys gave the appearance of being tough and of managing, feelings of loneliness and longing often lay beneath the surface. This impression was confirmed when two of the authors met some Afghan boys in a park in Belgrade. After telling us about their hard journey and how they had managed, we asked whether they missed their parents, and they responded by starting to cry.

Many had lost one or both parents. They sometimes expressed sadness but seemed emotionally distant to the loss of loved ones. This seemed to be a necessity during the harsh conditions they had to endure, with sparse contact with loved ones. Habdeh told how difficult it was when the surviving members of his family had been spread to different countries. While waiting for an answer to his asylum application in Norway, he told of how his role in the family had changed:

*Both my parents have passed away. My mother was ill and died in 2010. My father was ill and died in 2013. I am the oldest in the family*.

Habdeh had seven siblings, who all lived in Aleppo with the extended family before the war. Now they were separated, four in Syria and four in various countries in Europe. He expressed how his role as the eldest boy, replacing his father, was emotionally challenging to handle.

### Responses to Self: Who Am I Here, and Who Can I Be?

The participants had to leave their habitual sociocultural contexts, where they could have expected a reasonably predictable course of life, where relationships with others in the community would have helped or hindered their desired development. How they were looked upon and responded to by their primary family members, peers, teachers, and others should have been guiding them in the development of their identity. Thus, their flight deprived them of the sociocultural settings, which normally would have represented a frame of reference for developing their identity. Below, we will explore the ways in which the participants expressed their views about themselves, the responses they experienced, and how these have affected their self-image.

What emerged in the interviews was that many had a negative view of their life and of themselves, and that this was related to a lack of good and meaningful experiences, combined with many degrading encounters. Ahmad, a 16-year-old boy from Afghanistan, exemplified such experiences:

*When I was seven, I lost my mother and those were bad moments for me. Honestly, I have not had any good experiences in my life. So far, I have not had a good moment* (Interviewed in Serbia).

Salma, a 20-year-old woman from a minority group in Iraq, gave the following answer to the question of whether anything good had happened to her before or during her flight:

*No, they (good experiences) do not exist. I do not think anything nice happened to us, only that my mother and my brothers are with me (Interviewed in Serbia).*

Others, however, had preserved their wishes and dreams for the future that are normal for this age period, though they were often expressed in more general ways and often referred to the time before flight. They were, nonetheless, still active in their minds, and many expressed angry oppositions to the conditions they experienced in the asylum centers and an unwavering hope that things may get better. Amir, an 18-year-old boy from a minority group in Afghanistan, said:

*I loved living and studying a lot, and studying was my greatest wish. Apart from studies, I did not think of anything else. I wanted to finish high school, to enroll in college, I really wanted to enroll in some technical college to be an engineer* (Interviewed in Serbia).

Similarly, Samir, a 17-year-old boy from Afghanistan, talked about his earlier dreams of the future and of how these hopes were still vivid in his mind:

*I would rather go to school and learn the craft. I loved working with computers. I did photos with computers, technology, and stuff. I had great visions about my future, I loved sports a lot, I played football. I would like to be an athlete, to continue to play football, to continue to be a photographer and to continue and complete my studies* (Interviewed in Serbia).

Many participants talked about how they thought they were going to be received as refugees; of being welcomed and recognized as a human being in need. The need for recognition is normal for any human being, but especially important for young people. However, the experiences associated with this were often brutally disappointing. Ahmad, a 16-year-old boy from Afghanistan, elaborated:

*When I left Afghanistan, I thought when I came to European countries that they would respect and help us and look at us as human beings. We left our country because our lives were in danger. We thought that European countries would respect our human rights, but we see that here we are nothing to them and they do not respect us, as if we were not human* (Interviewed in Serbia).

During flight, several expressed some hope for the future, although often indirectly by talking of their plans for education when they were in their home country. The experiences of those interviewed in Norway showed that they had problems understanding how to interpret and manage their situation at the asylum centers, such as who they were, how they were perceived by others, and what developmental possibilities they had.

Some clearly expressed that they were in a dead-end situation. Shadi, an Iranian man now aged 24, came to Norway 8 years ago, aged 17. His application was rejected but he had no possibility to return to his home country. He felt totally helpless and did not know what to do or how to live his life. He said simply: “I have no capacities that can help me,” describing how in his present situation he saw no future possibilities and that he had no personal resources that could help him. It expressed the confused image he had of himself: “Who am I here, what can I be?”

Similarly, Hédi, a 24-year-old boy from an African country, felt he had become pacified and could not find the resources within himself to manage his situation. He had great difficulties managing his insecurity and anxiety while waiting for the decision on his application in Norway. Referring to his former life, he said he had happiness there, he could work and earn money:

*Here, I cannot earn anything, I have to “take” money from others. (…) I feel my life is meaningless. I am a person without any success. (…) What is the meaning with my life? Animals eat food and sleep. (…) What can I do? I feel hopeless … I feel hopeless. What can I do here?* (Interviewed in Norway).

Feelings of hopelessness and worthlessness, and for many an unclear image of who they were or could be, dominated the interviews of the participants in Norway. The context of waiting and not being able to do anything, of no school or of not being allowed to work, made life unbearable for many of the participants.

Another theme that came up in connection with identity was age. Many of the participants in Norway had been tested to verify their age and in order to determine whether they were to be treated as adults (above 18) or not. If the test results concluded that a person was over 18, it implied being moved to an asylum center for adults with considerably fewer facilities and opportunities. Many described the feeling of not being believed when they gave their age, combined with a sudden change from being treated as a minor to being treated as an adult, losing sources of care and follow-up. Who they were, their identity, was suddenly changed by a decision made by external authorities. As a result, some became passive and lost hope, but others strongly opposed the decision and saw this as a problem with the system. Yousuf, supposedly 18 years old from Afghanistan, said that his application had been rejected but that he had appealed, and he described the waiting time as extremely pacifying, worse than being in a war zone. As a result of the age test, he suddenly had become another person, deprived of his life as an adolescent in the center for minors, and now he was angry and confused:

*My biggest problem is that I am placed on this adult center (…) I have undergone such an age test. (He is aware of the uncertainties of the age test). How does this machine know my age? My mother knows* (Interviewed in Norway).

Abdi, a boy from Ethiopia, had been tested for his age and was found to be 18 years old, and consequently was transferred from a center for children to a center for adults:

*First, I went to school, then I got two times rejection of my application. I was then not allowed to go to school. And then they insisted I was more than 18 years old. That was a lie, I said I was underaged. That is why I was denied permission to stay, I did not like being a human here, only living (…). And, I have simply lost my taste for life now. I am not looked upon as a human being (…) I do not live like a human being here. Demoralized (pause), only living. (…) It is only dark, I am only siting here. (…) There is no difference from the dictatorship at home. (…) Youth in my age do many things in their life and they are in process of changing their lives and participating in society. So I have no hope, I cannot do anything, just sit and think* (Interviewed in Norway).

In the interviews, the participant’s view of themselves as worthless with no possibilities came repeatedly to the fore. Habdeh from Syria talked about the immense hardships he had endured during his lonely flight, which started when he was 14 years old. Now aged 18, he lived in an asylum center waiting for the decision on his application, feeling utterly lonely and with no sense of direction in his life. At the time of the interview, he was considering suicide as a possibility for change:

*I think about my family and how they are doing. There is worry and fright all the time. Has anything happened with them (…) you think all the time. My head works all the time. And I think about myself and my situation. I have not got A permit to stay. Maybe I will be rejected, what will happen then? (…) I try to sort my thoughts. I think about my future, but nothing is clear yet, about my future. (…) Life here at the center is very, very boring. One hate being here. One gets tired of the clothes one has. Thinks all the time. One wants to commit suicide* (Interviewed in Norway).

Habdeh went on to describe how he tried to give himself some hope by thinking that maybe he would get a residence permit the next day, and then maybe be able to start something. But all in all, he perceived his situation as almost unbearable, as everything was put on hold. His life in the asylum centers (he had been to several) was experienced as a lonely struggle and as a life with no care. He expressed profound confusion over how he could be active and develop, and who he could become. Since he saw no possibilities, suicide came to his mind as a solution.

What we see in these quotes is young people who are in situations of serious conflict regarding how they should view themselves based on the responses they receive from external others. Some feel resignation and sink into passivity, but others, like Yusuf, manage to keep a more positive image of themselves as people who can achieve things and make something of themselves.

The participant’s experiences of flight and of how they were received tested their endurance. Those interviewed in Serbia were on their way and tried to cross borders and find a way in spite of great obstacles. For those interviewed in Norway, the conflict between hope and hopelessness was clearer, and many described an unbearable feeling of just waiting, just “being in wait” as one put it. Many felt pressured into a passive position with few possibilities of being able to use their resources, while some felt they no longer had the capacity to do anything. In some cases, this could lead to thoughts of suicide as the only active possibility.

## Discussion

The narratives in this study were related in two different situations: during flight and in reception centers after arriving in Norway. They were told by young people, most of whom were adolescents when they started their refugee journey. The stories based on experiences in their homeland were similar, describing how war, persecution, harassment, and serious losses made it impossible for them to live in their country. The recent takeover of power by the Taliban in Afghanistan confirms their descriptions of the dangers experienced in regions, where the Taliban has pursued its campaign of terror for many years.

What was conveyed during flight in Serbia differs in some respects from what was conveyed in Norway. The participants in Serbia were still on their way, and even though they encountered severe difficulties and were mistreated by smugglers and police/border guards, they had some hope of reaching their goal, as exemplified by their repeated attempts to cross borders. Among those who were in Norway and were waiting for decisions or had been rejected, the tone and quality of their stories was different, often characterized by hopelessness, confusion, worry, and a sense of dislocation. In a previous work, we analyzed how living in inconclusive life situations that unfold in the context of a bewildering, bureaucratic system that is difficult to understand or influence causes feelings of confusion and powerlessness (Sagbakken et al., 2020). Being left in a situation of insecurity and unpredictability, often for several years, distorts people’s perception of time when they lose the ability to visualize their future life. This troubled perception of time was experienced as a continuation and reinforcement of unsafe and dehumanizing pre-flight and flight experiences.

### Time and Development

Central in developmental processes is the ability to link the past to the present, and the present to the future. This is especially true in the adolescent and young adult phases. In fact, a central task in these phases is to imagine future possibilities and to find what grounds or capacities one has developed to manage whatever one sees as possibilities. One needs to be aware of one’s desires and wishes, which often can be unclear and can provoke anxiety, and to find out which wishes and desires one is capable of realizing, which ones are acceptable, and which ones can be realized within the sociocultural context in which one lives (Erikson, 1963; Syed and Fish, 2018; Greene, 2021).

As previously described, adolescence implies a major reorganization on biological, psychological, and social levels, and is a period where the need for contextual stability (family, social context) is critical for being able to manage the turmoil and internal upheaval one goes through. The psychosocial context represents a frame of reference for orienting oneself in time and space. To achieve this, one needs some stability in order to connect the past and the present with the future and to imagine what one can develop to become or how one’s identity can develop. This difficulty in imagining a future with possibilities seemed to create a sense of uncertainty and meaninglessness, which caused impairment in the sense of reality, whereby cognitive organization of perceptual impulses became extremely demanding due to difficulties in distinguishing the important from the unimportant (Sagbakken et al., 2020).

Thus, when the context of development collapses, the consequences may be serious for managing development tasks. Within a context, where external and alien forces during flight (cheating and unpredictable smugglers, brutal behavior by police and border guards, and human rights violations) and the bureaucratic, unpredictable jungle experienced on arrival, the time frame of development becomes extremely difficult to maintain (Christensen and Lindskov, 2000). Without reliable guidance, these young people must manage by themselves and make up for whatever the context did not provide by trying to organize their mental life in an effort to imagine and foresee what may come.

In the interviews, we heard how many tried to think of education, but again and again they returned to how all hopes were dashed in their home country. As Habdeh said: “There was war in Syria. There was killing, beheading, and no school. We are a lost generation.” He expressed a general trend among our participants. They fled from extreme conditions with no or few developmental possibilities and found themselves in a situation of limbo, with the hopeless feeling of being subjects of impersonal forces which seemingly did not care for them.

### Tasks to Solve: Erikson’s Stages

Identity formation is seen as the primary developmental task during adolescence (Erikson, 1963, 1968). Erikson explored nuances and variance in these developmental processes and how social, historical, and political contexts interact with personal development (Syed and Fish, 2018). Healthy identity implies, in this perspective, sense of temporal and contextual integration and identity can be understood as an integration of the numerous, possibly conflicting, and aspects of individual lives (Akhtar, 1995; Johansen and Varvin, 2020). In Erikson’s eight developmental stages, development in each stage is dependent on how successful or unsuccessful the previous stages have been. Each stage implies a psychosocial crisis in the sense that the expectations from the environment demand reorientation and an expansion of the interpersonal and psychosocial world (Erikson, 1950). There is no deterministic relationship between the stages. Healthy development may ensue even if earlier stages have been marked by much stress or trauma, especially if interpersonal relations can give support and promote healing and resilience (Erikson, 1995). Thus, the context and the person mutually influence each other (Sameroff, 2010). To manage the psychosocial crisis in each developmental stage, there cannot be too big a discrepancy between the capacities of the person and the potential of the ecological environment.

At the core of the adolescent crisis lies the tension between developing a more stable identity and identity confusion (Erikson, 1963). The interpersonal relationships in which identity develops need to be stable enough to avoid identity diffusion and confusion. Early hardships and potentially traumatizing experiences in childhood may lower resistance and the ability to cope during later hardships (Opaas and Varvin, 2015). There is thus the possibility that participants in our research have been especially vulnerable to the hardships they encountered during and after flight, and that these may have contributed to the hopelessness described by, for example, Salma as follows: “No, they (good experiences) do not exist. I do not think anything nice happened to us, (…).” Salma expresses resignation, as if she had acquired the identity of a person who has been and will be treated badly.

Following Erikson and others, problems in earlier developmental stages influence how later developmental crises are solved (Erikson, 1964; Lilly and Lim, 2012; Mikulincer and Shaver, 2015; Opendak et al., 2017). We do not have much specific data on early childhood in this study, but research on other groups of refugees has revealed abundant early childhood hardships (Opaas and Varvin, 2015). During later hardships and upheavals, the consequences of earlier traumatization may be actualized in the form of catastrophic anxieties, and the present may be perceived as confirmation of the early experiences of danger and unpredictability (Varvin, 2003; Rosenbaum and Varvin, 2007). Early primitive defenses may reappear, such as splitting and projective identification, leading to increased confusion, identity diffusion, and lack of any possibility to organize future perspectives in the mind (Henningsen, 2005). Many of the participants expressed hope, but it is a hope that cannot be anchored in a defined future experience. The participants experienced the waiting for something that would define their lives for better or worse, but with limited possibilities to influence the outcome: one can try again to cross a border or send a new application, but those who decide are outside their sphere of influence (Biehl, 2015; El-Shaarawi, 2015; Horst and Grabska, 2015).

A fundamental resource for displaced people is hope for the future, and thus the possibility of “future-figuration” (Akhtar, 1999; Artero and Fontanari, 2021), a possibility that is severely reduced among the participants in this study.

Habdeh’s comment on the lost generation represents an interesting reflection on the loss of this resource, a situation that seemed typical for many of the participants in this research: no possibilities at home, and an extremely insecure future. A future directedness as a driver for development is seriously disturbed.

### What Kind of Identities Can Develop in the Refugee Context: The Limbo Experience

In a previous publication (Sagbakken et al., submitted), we described the experience of rejected asylum seekers as one of being in limbo, referring to their experiences of endless waiting. In this context, the word “wait” lost its meaning because for them there was nothing to wait for (Bjertrup et al., 2018). The term “liminal phase” is often used to refer to the stage in a “rite of passage,” where the subject is on the threshold to the new and partly unknown (Turner, 1964, pp. 4–20). It is often described as the transition phase between two stages where the next is more or less known, like a “passage of transition” between youth and adulthood (Turner, 1964). Being in limbo, on the other hand, is a situation of being stuck, with limited possibilities to move forward or develop into the next phase. To be in limbo, according to Collins dictionary, means to be “in a situation, where you seem to be caught between two stages and it is unclear what will happen next,” or to be in an “imaginary place for lost, forgotten, or unwanted persons or things” (Collins Dictionary, n.d.), which is similar to the feelings of many of the participants in our study as well as in others (de Zulueta, 2011; Nimführ and Sesay, 2019).

Adolescence can be described as a liminal phase, and different cultures provide possible outcomes that offer possibilities. The participants in this research had all fled to arrive at some place with a wish for new possibilities that were not present at home. Their flight can thus be seen as an extended liminal phase, where all kind of hopes and desires were activated, but with a realization that there were limited possibilities of realizing any wished-for plans. They had as a rule been backed by their families and filled with hopes and expectations. Their will to fulfill these hopes and obligations and to help their families were apparent in many interviews. The situation of the participants was thus complex: they were in the middle of a complicated developmental phase involving bodily changes, mental reorganization, and establishment of capacities for both intimate and social relationships. At the same time, they strived to fulfill the tasks and hopes given them by their families, or what they themselves imagined was important for their kin. In other words, they strived to establish identities both as grown-up and relatively independent individuals and as members of their group or family, with the obligations this imposed on them. In other words, a complicated, multidimensional, mental, and sociocultural situation, where they have to deal with biopsychological development, relationships with their family and group, cultural pressures, and the flight/exile situation (Rosenbaum and Varvin, 2007; Rosenbaum et al., 2020):

Situations of stress, crisis, or traumatization challenge one’s capacity to cope and resist and creates a situation where resilience can be activated if enough early resources have been established (Alayarian, 2007). The possibility to construct meaning of experience is of overriding importance. It is in a person’s relationship with cultural discourses that meaning can be constructed from experience. This gives the person reason to believe that there can be a continuation, and thus create a time dimension. It influences relationships in the group, where identity is shaped by the reciprocal relationships with others and where one gradually learns who one is and who one wants to be, based on responses from others. The cultural umbrella thus safeguards a meaning-producing dimension that helps a person endure crisis and hardship (Obeyesekere, 1990).

Without these relational contexts, identity formation becomes difficult. Participants in this study have experienced major upheavals and breakdowns in their development so that to a certain degree they lack what could have served to support and guide their identity development. What the participants described was a replacement of relational contexts with peer groups (fellow refugees, some of whom were dangerous), with smugglers who were notoriously dangerous and unreliable, and with border guards/police who looked upon them as intruders or strangers who were to be rejected or thrown out. Additionally, they described the lack of a cultural, meaning-producing system with its moral values and implications. What the participants met was a chaotic, unpredictable context and an absence of common, moral guidelines.

The formation of identity is always a process and is always dependent on responses from others and being seen as an outcast or as dangerous or of no worth marks people’s identity development. Thus, being a young refugee in these contexts severely disturbs both development of identity and the sense of being of worth.

Follow-up studies on refugees in receiving countries show variable outcomes regarding mental health and quality of life, but many show resilient development (Vaage et al., 2010; Bogic et al., 2015). There is, however, a tendency of long-term mental health problems (Opaas et al., 2020), especially since support and treatment tend to come late and be insufficient (Pumariega et al., 2005; Opaas and Varvin, 2015).

### “To Be in Wait”: Passivity vs. Aggression

This expression by one participant conveys development of an identity characterized by passivity. It expresses an empty space where no one is there to see the person, affirm their existence, give some hope that waiting may have an end and to express personal hope for the person. Being in situations where feedback on who one is comes from people who want to exploit, reject or harm you creates a special situation of uncertainty, not only on a social level (Horst and Grabska, 2015), but also on an internal level. Development of one’s identity is always an interaction between one’s involvement in a group and the reorganization of one’s internal world. Culture becomes part of the mind through these interactions, and when one lives in an extremely dangerous and aggressive world, this may be internalized, creating internal instability and often a tendency to identify with the aggressor (Hirsch, 1996). One internalizes the relationships one is in to manage and survive in a dangerous world. This is the background for what is called the externalizing behaviors that can be seen among young refugees (Fazel et al., 2012). Identifying with the aggressor protects against the helplessness one experiences at the hands of the perpetrator (Frankel, 2004). This was very clear when interviewing the participants in the park in Belgrade. It took time to build trust, the participants behaved for a long time as if the researchers were allied with the police and treated them accordingly with suspicion and animosity. Thus, we may interpret this as a passive-aggressive attitude that helped them manage in unclear situations.

The same has been observed in traumatized refugees in treatment. Long periods in the treatment process may be characterized by passive-aggressive attitudes (Varvin, 2003). The task in these treatments is then to activate something good in the internal world of the patient, a relationship with some good and caring inner object. What is attacked and often destroyed during atrocities like torture and serious maltreatment is this inner link to an empathic other (Laub and Podell, 1995; Laub, 1998), which is essential to the development of a subjective sense of a stable identity (Erikson, 1968). The mistrust and skepticism, we saw in our participants can be seen as a survival strategy during extreme circumstances (Varvin, 2003). What is needed then, to help young refugees both during and after flight, is help with establishing inner security, which happens through mobilizing inner resources in good inner relationships. Early interventions and caretaking after traumatization and hardships may thus be of importance for mental health and social functioning in the long run (Keilson and Sarpathie, 1979; Steel et al., 2011). This leads to the questions of how refugees should be cared for and what interventions and treatment they need.

### Interventions/Treatment

Due to the hardships and potential traumatizing experiences during a vulnerable phase in their lives, it is to be expected that many will develop psychological problems in the long term (Henley and Robinson, 2011; Vervliet et al., 2013; Jensen et al., 2014). We have shown that there are many uncovered psychosocial needs in this group, both during and immediately after flight. As a rule, these needs are not met and any organized and planned help is scarce.

Based on the findings of this research and other sources (Jensen et al., 2014; Thommessen et al., 2015; Keles et al., 2016), we hold that help should be provided as early as possible and at any stage in the flight. It should be comprehensive and flexible (i.e., it should include various forms of health care, mental health care and psychosocial interventions as well as legal protection, security measures and addressing basic needs), be easily accessible, sensitive, and tailored to trauma and torture victims, and be well designed to tackle obstacles that are well recognized in provision of services for asylum seekers, such as low awareness of services, linguistic barriers, and differences in cultural perspectives and service preferences (Satinsky et al., 2019). Interventions should also take into account that the social functioning of individuals who have experienced traumatization and torture can be impaired (Jović, 2017). They should cut across traditional medical and mental health care and social services, be tailored to cover as wide an area as possible and have an interdisciplinary and intersectoral approach. According to IASC^3^, “the composite term mental health and psychosocial support is used in this document to describe any type of local or outside support that aims to protect or promote psychosocial well-being and/or prevent or treat mental disorder” (IACSC, 2007).

It is already recognized that there is a “need for a comprehensive, well-planned approach at the different stages in the refugee journey” (Varvin, 2021, p. 453) during flight, in refugee camps on the route (which are often improvised crowded shelters with inadequate accommodation), in asylum and reception centers on arrival, all the way to the (ideal) completion of the asylum procedure, settlement in a new country and new home, and adjustment to a new physical, social and cultural environment. These services will depend on the needs of refugees and should be adapted to the flight regarding safety concerns and medical consequences of physical and psychological stress. After resettlement there will be a need for comprehensive, specialized services to prevent chronic sequelae.

A model for such help and support during flight was developed in Serbia in 2015 by International Aid Network, Center for Rehabilitation of Torture Victims (IAN/CRTV) for refugees passing through the country and in need of basic help and support. Mobile teams were organized comprising a medical doctor and a nurse to attend to refugees’ physical and medical needs using a set of interventions known as medical first aid (MFA). The teams also comprised a psychologist, responsible for providing psychosocial first aid (PFA), and a translator (Jovic, 2018). Guidelines for brief psychosocial interventions for refugees were developed that included:

1. Safety, at a time when it was important to determine whether the person is at real physical risk of falling victim to peer violence, human trafficking, sexual abuse, or forced prostitution.
2. Basic needs (food, hygiene items, or shelter).
3. Legal protection (support with registration procedures, asylum applications, and their rights in both cases).
4. General health status/needs (physical health, chronic disorder, or physical trauma).
5. Assessment of mental health needs, psychological and psychosocial support, and treatment and rehabilitation.

As refugees are now mainly accommodated in reception centers in Serbia and on the Balkan route, it is highly probably that their mental health needs will come to the fore, due not only to high incidence of posttraumatic responses, depression, suicidal behavior, psychosis, family problems/pathology, but also to conditions in camps that are harmful to health (Varvin, 2021). Still, organizing well-structured treatment is a challenging task on account of the very basic services provided by the health care sector and frequent changes in accommodation.

Few studies have evaluated early prevention and treatment programs. Studies assessing community-based interventions, such as outreach programs, workshops, train-the-trainer models, refugee employment schemes and mentoring programs focused on self-help, inclusion, empowerment and advocacy, consistently found some reduction in symptomatology in participants (Williams and Thompson, 2011), and a long-term follow-up of early intervention showed promising results (Drozdek et al., 2014).

Comprehensive preventive programs are therefore a necessity, especially for vulnerable groups such as unaccompanied minors and for adolescents in general. Based on clinical/practical experience, we believe that the above guidelines outlined by IAN/CRTV will have positive effects on refugee groups as well as on the community they function in, as indicated in review of Williams and Thompson (2011). There is thus a need for further systematic studies.

## Conclusion

Young people experience a radical break in their development when they embark on their refugee journey. They flee from war, persecution, and often from countries where the infrastructure has broken down. They bear the double burden of solving developmental challenges, while trying to adapt to dangerous conditions during flight and incomprehensible bureaucratic systems in the country in which they seek asylum. Many feel obligated to their families to succeed and be able to support their family at home. Many have suffered severe losses and have been traumatized. Their need for safety and to able to plan is severely hampered, and their developmental needs are generally not met.

Furthermore, young refugees are at risk of developing mental health problems and of having difficulties adjusting. It is therefore argued that support and help should be set in as early as possible, preferably already during flight, and that their life in reception countries should be supported and treatment (mental and somatic) provided when necessary, regardless of their status in the asylum system. They should be seen as young people in important developmental phases, and interventions and help should be adapted to this situation. Suggestions for how this could be organized are provided above.

The participants in this study were selected from a larger study in which 178 people were interviewed either in a reception center or in an outside location. The focus on adolescents and young adults in the larger study facilitated a study of a typical group in the chosen contexts (Serbia and Norway) at the time. The in-depth quality of the interviews and the fact that we chose the more comprehensive interviews strengthen the validity of the findings. The specific context for the interviews, e.g., that most of the participants were in a desperate situation, also imposes limits in that it influenced how the narratives were constructed at the time of the interview. However, we took their specific situation into consideration when performing the analysis, and participant observations combined with notes from the interviews (body language, expressions of emotions, etc.) helped validate what was expressed verbally.

As this is one of few studies to focus on the developmental aspects of being a refugee, we believe it may pave the way for more studies that focus on refugees as ordinary human beings who need to take their developmental health into consideration, regardless of the social context they find themselves in.

## Data Availability Statement

The raw data supporting the conclusions of this article will be made available by the authors, without undue reservation.

## Ethics Statement

The studies involving human participants were reviewed and approved by Norwegian South-East Regional Committee for Medical and Health Research Ethics (2016/651), The Commissariat for refugees and migration and in collaboration with the Center for Rehabilitation of Torture Victims (CRVT) and International Aid Network (IAN). The patients/participants provided their written informed consent to participate in this study.

## Author Contributions

SV and MS developed the study design and conceptualization and completed interpretations of the analysis. MS, SV, and IV conducted the interviews. All authors participated in process of developing the data analysis. SV developed the main text of the paper. All authors reviewed the results and approved the final version of the manuscript.

## Funding

This research has been supported by Department of Health Sciences, Oslo Metropolitan University, Faculty of Philosophy, University of Priština (Kosovska Mitrovica), and the Center for Rehabilitation of Torture Victims, IAN, Belgrade.

## Conflict of Interest

The authors declare that the research was conducted in the absence of any commercial or financial relationships that could be construed as a potential conflict of interest.

## Publisher’s Note

All claims expressed in this article are solely those of the authors and do not necessarily represent those of their affiliated organizations, or those of the publisher, the editors and the reviewers. Any product that may be evaluated in this article, or claim that may be made by its manufacturer, is not guaranteed or endorsed by the publisher.

## Footnotes

^1^There are cultural and social differences in how the age periods for adolescence and young adulthood are defined. Adolescence is often from 12–13 to 18 years and young adulthood from 18 years up to 30+. In this paper, we decided to use 13–18 years for adolescents and 18–28 years for young adults. The interviews were selected based on what we judged was the participant’s psychological age, partly independent of their chronological age.

^2^All names are fictitious.

^3^The Inter-Agency Standing Committee is an inter-agency forum for coordination, policy development and decision-making involving the key UN and non-UN humanitarian partners.
